# Multicenter study of the performance of NTM Elite agar for the detection of nontuberculous mycobacteria from patients with cystic fibrosis

**DOI:** 10.1128/spectrum.02736-23

**Published:** 2024-08-28

**Authors:** Emmanuel André, Natalie Lorent, Kurt Beuselinck, Susanne Deiwick, Lieven Dupont, Johanne Gafsi, Lies Laenen, Lise Raymaekers, Pascal Van Bleyenbergh, John D. Perry, Barbara C. Kahl

**Affiliations:** 1 Laboratory Medicine Department, UZ Leuven University Hospitals, Leuven, Belgium; 2 Department of Microbiology, Immunology, and Transplantation, Laboratory of Clinical Microbiology, KU Leuven, Leuven, Belgium; 3 Respiratory Diseases Department, University Hospitals Leuven, Leuven, Belgium; 4 University Hospital Münster, Institute of Medical Microbiology, Münster, Germany; 5 BioMérieux Global Clinical Affairs, Marcy-l’Etoile, France; 6 Microbiology Department, Freeman Hospital, Newcastle upon Tyne, United Kingdom; University Paris-Saclay, AP-HP Hôpital Antoine Béclère, Service de Microbiologie, Institute for Integrative Biology of the Cell (I2BC), CEA, CNRS, Clamart, France

**Keywords:** nontuberculous mycobacteria, cystic fibrosis, mycobacterial culture

## Abstract

The performance of a novel selective agar was evaluated against the performance of conventional mycobacterial cultures, i.e., a combination of the mycobacterial growth indicator tube (MGIT) with Löwenstein-Jensen (LJ), for the detection of nontuberculous mycobacteria (NTM) in sputum samples from people with cystic fibrosis (pwCF). Two hundred eighty-three sputum samples (231 fresh sputum and 52 spiked sputum) from 143 pwCF were collected. They were inoculated without prior decontamination on NTM Elite agar (30°C ± 2°C for 28 days) and inoculated on both MGIT and LJ (35°C–37°C for 6–8 weeks) after N-acetyl-L-cysteine-2% sodium hydroxide decontamination. NTM were identified by Matrix-Assisted Laser Desorption Ionization/Time of Flight Mass Spectrometry and/or PCR, and whole-genome sequencing. A total of 67 NTM were recovered overall by the combination of all culture media. NTM Elite agar allowed the recovery of 65 NTM (97%), compared to 22 for the conventional MGIT and LJ media combination (32.8%), including 22 NTM for MGIT (32.8%) and 3 NTM with the LJ medium (4.5%). For *Mycobacterium abscessus* complex, the sensitivity of NTM Elite agar was 95% compared with a sensitivity of 30% for the conventional MGIT and LJ media combination. Overall, 17.3% of cultures on NTM Elite agar were contaminated with other micro-organisms vs 46.3% on MGIT and 77% on LJ. This study shows that the novel selective agar (NTM Elite agar) significantly outperforms the conventional MGIT and LJ media combination in terms of sensitivity, selectivity, and ease of culture, without the requirement of an L3 laboratory.

**IMPORTANCE:**

Nontuberculous mycobacteria (NTM) are significant pulmonary pathogens in patients with pre-existing structural lung conditions such as cystic fibrosis, bronchiectasis, or chronic obstructive pulmonary disease. *Mycobacterium avium* complex and *Mycobacterium abscessus* complex (MABSC) are the most frequently isolated organisms. Compared to the recommended culture method for NTM, which combines solid and liquid culture media, NTM Elite agar enables a faster/easier diagnosis and speeds up identification and susceptibility testing as the final reading is at 28 days instead of 6–8 weeks for the conventional mycobacterial cultures. In addition, for the NTM Elite agar, no decontamination stage before inoculation is necessary, unlike the conventional mycobacterial cultures. NTM Elite agar is derived from a formulation of medium adapted to rapidly growing mycobacteria (RGM). The medium enables the growth of RGM while suppressing other flora. It is supported with published clinical data showing the benefits of this medium.

## INTRODUCTION

Nontuberculous mycobacteria (NTM) are well documented as respiratory pathogens among people with cystic fibrosis (pwCF). Most commonly found NTM species in Europe and North America belong to the slow-growing *Mycobacterium avium* complex or the rapidly growing *Mycobacteroides* (*Mycobacterium*) *abscessus* complex (MABSC) (1–3). MABSC has been associated with accelerated lung function decline (4) and may preclude pwCF from lung transplantation because of suboptimal outcome. The US Cystic Fibrosis Foundation and the European Cystic Fibrosis Society recommend cultures with both solid and liquid media, using an automated growth detection system, for a minimum of 6 weeks for the detection of NTM in respiratory tract samples of patients with CF (5). A decontamination using standard N-acetyl-L-cysteine-2% sodium hydroxide (NALC-NaOH method) is recommended prior to culture (5). Nevertheless, NTM colonization and infection are under-reported due to other bacteria resisting the NALC-NaOH decontamination used prior to conventional mycobacterial cultures (6, 7), possibly resulting in masking of NTM, and partial inhibition of some strains of NTM by this decontamination process. A recent study using sputum samples from pwCF showed that optimal recovery of MABSC is achieved by culture on an appropriate selective agar without decontamination of sputum samples (8). This selective agar, called RGM medium, was previously described (9), and its performance was assessed during studies involving individuals with (10, 11) and without CF (11, 12). Industrialization of this selective agar medium was achieved resulting in the NTM Elite agar. The aim of this study was to assess the sensitivity and selectivity of NTM Elite agar (without decontamination) on sputum from pwCF compared to the conventional mycobacterial cultures combining a mycobacterial growth indicator tube (MGIT) and a Löwenstein-Jensen (LJ) medium following a decontamination step. In addition, an equivalency study was conducted that compared the performance of RGM medium and NTM Elite agar.

## MATERIALS AND METHODS

### Study design

The prospective multicenter study was conducted at two clinical trial sites, the University Hospitals of Leuven, Belgium and the Münster University Hospital, Germany.

### Patient samples

No additional samples were taken from patients for the purpose of the study. The study was approved by an ethics committee at each site and conducted in accordance with the Declaration of Helsinki, Good Clinical Practice, and applicable regulatory requirements. Informed consent was collected when required by the ethics committee for patients included in the multicenter prospective study.

A total of 283 sputum samples from 143 pwCF aged from 16 to 84 years old were collected from the two clinical sites from July 2019 to March 2020. In addition to fresh sputum samples (*n* = 231), contrived samples (*n* = 52) were included in the study to reach the required sample size. A total of 247 samples were included from the University Hospitals Leuven (195 fresh clinical samples and 52 contrived samples) and 36 samples from the Muenster University Hospital (only fresh clinical samples). All sputum samples from pwCF, with sufficient remnant volume (at least 0.5 mL) to allow additional culture on NTM Elite agar (bioMérieux, Craponne, France) compared to conventional MGIT (Becton-Dickinson, Sparks, USA) and LJ (bioMérieux, Craponne, France; or Enclit, Borna, Germany; or Becton-Dickinson, Sparks, USA) media, were consecutively included in the study. If a patient was sampled with at least two sputa at a single time point of the study, only one sample was included in the study. Samples for which the protocol could not be performed entirely were excluded from the study. Samples were tested on the day they arrived in the laboratory which was within 24 h of being taken from the patient. If samples needed to be stored overnight, they were refrigerated at 4°C.

### Contrived samples

A total of 52 sputum samples from patients with CF were spiked with NTM. These samples had to meet the following criteria to be spiked: samples with sufficient volume to perform all cultures (NTM Elite agar and conventional MGIT and LJ media) and allowing an additional volume to be contrived; negative smear microscopy for acid-fast bacilli; and negative PCR screening for *Mycobacterium* genus and *Mycobacterium tuberculosis* complex (MTBc). The laboratory-developed multiplex Quantitative Polymerase Chain Reaction (qPCR) used targets the 16S–23S rRNA internal transcribed spacer gene present in all mycobacterial species and the glycoprotein B gene of PhHV-1 (used as internal control). PCR reaction was performed using the Quanstudio Dx platform (Thermo Fisher Scientific, Waltham, Massachusetts, United States). Real-time PCR was carried out in a reaction volume of 30 µL containing 20 µL of the DNA extract, 7.5 µL TaqMan Fast Virus 1-Step Master Mix and a set of primers described in Table 1, using the following cycling conditions: 20 seconds at 95°C, followed by 45 cycles of 95°C for 3 seconds and 60°C for 30 seconds.

**TABLE 1:** Primers used for PCR screening of *Mycobacterium* genus and MBTc

Target	Primer/probe	Primer/probe sequences	Concentration (µM)
*Mycobacterium*	Forward primer^*a*^	5′-CACCTCCTTTCTAAGGAGCACC-3′	0.25
	Reverse primer^*a*^	5′-ATGCTCGCAACCACTATCCA-3′	0.25
	Probe	FAM-5′-CACACCCCACCACCAA-3′-MGB	0.1
	Probe	FAM-5′-ACACCCCGCCACCAA-3′-MGB	0.1
*M. tuberculosis*	Probe	NED-5′-ACCTGGAACAAGTCC-3′-MGB	0.2
PhHV-1	Forward primer^*b*^	5′-GGGCGAATCACAGATTGAATC-3′	0.1
	Reverse primer^*b*^	5′-GCGGTTCCAAACGTACCAA-3	0.1
	Probe	VIC-5′-TCCGCCACCATCTG-3′-MGB	0.1

^*a*^ Adapted from Roth et al*.* (13).

^*b*^ Adapted from Niesters et al. (14).

All samples were liquefied with a dithiothreitol-based agent (Sputasol, Oxoid, Basingstoke, UK) according to the manufacturer’s instructions. Then, a 2 mL volume was sampled from the liquefied sputum samples and spiked with 20 µL of a mycobacterial suspension [1.5 × 10^5^ Colony Forming Units (CFU)/mL]. The remnant part of the liquefied sputum sample was processed as a non-contrived sample and therefore underwent culture on LJ, MGIT, and NTM Elite agar without spiking to confirm the absence of mycobacteria. An additional volume of 20 µL of the same mycobacterial suspension was inoculated on Middlebrook 7H10 medium (Sigma Aldrich, Buchs, Switzerland; or Becton Dickinson, Heidelberg, Germany) and incubated without CO_2_ to control viability and purity of the mycobacteria suspension. The NTM isolates spiked into the sputum samples originated from bioMérieux stock collection or from the stock collection of the University Hospitals Leuven. Stock isolates originated from respiratory samples of pwCF or were from species usually recovered from respiratory samples of pwCF. Stock isolates that had been initially isolated on NTM Elite agar or RGM medium were not included in this study.

### Culture on NTM Elite agar

NTM Elite agar could be inoculated with one of the following processes: either sputum samples were inoculated directly on NTM Elite agar with a swab, or a volume of 100 µL was inoculated with a pipette and then streaked with a loop. Prior to that, non-selective liquefaction with a dithiothreitol-based agent (Sputasol) could be performed when deemed necessary (e.g., sample too viscous or low volume to homogenize the bacterial inoculum). Subsequently, NTM Elite agar plates were placed in a sealed plastic bag and incubated for 28 days at 30°C ± 2°C in ambient air. Plates were examined for growth after 4 and 7 days and then weekly. It is noteworthy that the interference of Sputasol on the NTM Elite agar plate was studied using a Sputasol suspension mixed with a bacterial suspension with a final Sputasol concentration similar to a clinical sample. Interference was assessed based on bacterial growth density and colony size. If either one of these parameters decreased on the NTM Elite agar plate but was not observed on the Lowenstein-Jensen control medium, it would have been deemed that the substance interfered with the NTM Elite agar medium. The interference of Sputasol was studied using eight nontuberculous mycobacteria and five non-mycobacterial organisms. No interference was observed regarding the use of Sputasol with the NTM Elite agar plate.

### Culture on conventional MGIT and LJ media

Sputum samples were subject to a decontamination and liquefaction step with N-acetyl-L-cysteine and sodium hydroxide-based agent as per sites routine, i.e., sodium hydroxide (3% for Muenster and 4% for Leuven) was dissolved with one vial of N-acetyl-L-cysteine powder for 15–20 min, then neutralization buffer was added, and the solution was centrifuged at 3,000 U/min for 20 min. Then, a smear of the samples was prepared, and staining of the smear was performed using Auramine O or Ziehl-Neelsen depending on the routine of each clinical trial site. The samples were then cultured on both liquid (MGIT) and solid media (LJ), as recommended by international guidelines (5). Both were incubated at 35°C–37°C for 6–8 weeks. The LJ medium was read at the same time as NTM Elite agar, i.e., at 4, 7, 14, 21, and 28 days, in addition to the final reading between 6 and 8 weeks. Readings could be postponed up to 2 days except for reading at 28 days and final reading. MGIT was automatically read by the BD BACTEC.

### Identification

Acid-Fast Bacillus (AFB) staining with Auramine O or Ziehl-Neelsen was performed on each type of colony to exclude non-mycobacteria. For colonies grown on NTM Elite agar, subculture was performed before identification if necessary (e.g., if only a few colonies were present). This subculture was performed on Middlebrook 7H10 agar (Sigma Aldrich, Buchs, Switzerland; or Becton Dickinson, Heidelberg, Germany) and incubated without CO_2_ if mycobacteria were suspected after microscopy or on blood agar if a contaminant was suspected after microscopy. For colonies grown on Löwenstein-Jensen medium, if mycobacteria were suspected after microscopy, a Middlebrook subculture was performed if necessary. MGIT-positive cultures were subcultured on Middlebrook agar. When microscopy indicated the growth of a contaminant on conventional MGIT and LJ media, no further action was required.

All colony types from NTM Elite agar and suspect mycobacteria colonies from conventional MGIT and LJ media were identified by Matrix-Assisted Laser Desorption Ionization-Time of Flight (MALDI-TOF) mass spectrometry (Bruker) and/or PCR GenoType Mycobacterium CM (Hain Lifescience) as per site routine. All mycobacteria were further identified by whole-genome sequencing (WGS) with Illumina paired-end sequencing technology (2 × 150 bases). A blast of the full *hsp65*, *rpoB*, and 16S genes was compared to public sequences available in NCBI/genbank. Then, phylogenic trees were performed in Geneious tool (Dotmatics) for *Mycobacteroides* genus and *Mycobacterium* genus based on *rpoB* and *hsp65* full genes. BLAST and phylogeny studies have been performed for all strains, regardless of the results. When BLAST gives close similarity between different taxa, a phylogeny study was used to identify a species. For phylogenetic trees, bootstraps of 80 at the last divergence node were considered robust enough to identify the strain. If this criterion was not reached, those strains could not be identified and, therefore, were classified as “unspeciated” strains.

### Statistical analysis

All NTM isolates, i.e., those recovered from 231 fresh clinical samples and from 52 spiked samples, were considered to assess the sensitivity of each method. For assessment of selectivity, organisms other than mycobacteria were quantified from the 231 fresh clinical samples and from the un-spiked portion of the 52 samples used in the spiking experiments. The sensitivity of NTM Elite agar and conventional MGIT and LJ media combination was compared based on their confidence interval (α = 5%). The same method was used to compare the NTM Elite agar selectivity to conventional MGIT and LJ media combination selectivity.

### Equivalency study between NTM Elite agar and RGM medium

The formulation of the RGM medium had been slightly adjusted to allow industrialization. Even though the formulations were very similar, RGM agar is a complex medium with 24 distinct ingredients as documented in Preece et al. (9). Therefore, there is a lot of scope for variability when these are sourced from different suppliers. An equivalency study was performed to confirm that both formulations were equivalent. The comparison of the performance of NTM Elite agar and RGM medium was conducted at The Newcastle upon Tyne Hospitals NHS Foundation Trust, UK. RGM medium was prepared as previously described (11). Aliquots of sputum (0.5 mL) were retained from 30 distinct individuals with CF after routine laboratory processing of the samples was complete. Samples were tested on the day they arrived in the laboratory which was within 24 h of being taken from the patient. If samples needed to be stored overnight, they were refrigerated at 4°C. The samples, which had been liquefied using Sputasol, were from pwCF with no history of NTM infection. One hundred microliter aliquots of each sample was inoculated onto RGM medium and NTM Elite agar.

The remaining 300 µL of each sample was inoculated with 30 µL of a suspension of NTM prepared at 1.5 × 10^4^ CFU/mL. A different strain of NTM was inoculated into each of the 30 samples. The NTM strains included: MABSC (subsp. *abscessus*, *n* = 6, subsp. *massiliense*, *n* = 3, and subsp. *bolletii n* = 1), *Mycobacterium chelonae* (*n* = 3), *M. avium* (*n* = 3), *Mycobacterium intracellulare* subsp. *intracellulare* (*n* = 3), *M. intracellulare* subsp. *chimaera* (*n* = 3), *Mycobacterium colombiense* (*n* = 1), *Mycobacterium gordonae* (*n* = 3), *Mycobacterium fortuitum* (*n* = 2), and *Mycobacterium simiae* (*n* = 2). One hundred microliter aliquots of each sample was inoculated onto RGM medium and NTM Elite agar. All plates were incubated at 30°C ± 2°C for 28 days and read after 4 days, 7 days, and then weekly. Any colonies recovered on the two media were identified by MALDI-TOF mass spectrometry.

## RESULTS

A total of 231 fresh sputum samples and 52 spiked samples from 143 pwCF were collected from July 2019 to March 2020. Among the 231 non-contrived samples, 46 samples (20%) were processed with Sputasol because of viscosity (*n* = 41) or low volume (*n* = 5). During the study, 21 NTM were recovered from 19 patients with cystic fibrosis: one patient had two samples out of three containing one mycobacteria each, and another patient had two mycobacteria recovered from a single sample. In addition, 46 NTM were spiked into sputum samples from patients with cystic fibrosis.

Of the 21 NTM isolated from fresh sputum samples during the study, NTM Elite agar allowed the recovery of 20 NTM, i.e., a sensitivity of 95.2% [95% CI (95% CI) 76.2–99.9] compared to six recovered on conventional MGIT and LJ media, i.e., sensitivity of 28.6% (95% CI 11.3–52.2), including six NTM from MGIT (28.6% [95% CI 11.3–52.2]) and one NTM from LJ medium (4.8% [95% CI 0.1–23.8]) as described in Table 2.

**TABLE 2:** Nontuberculous mycobacteria from 231 fresh sputum samples from 143 pwCF recovered on NTM Elite agar and conventional mycobacterial cultures (LJ and MGIT)

	Total number of NTM recovered	28-day culture on NTM elite agar	42- to 56-day conventional mycobacterial cultures (MGIT and LJ)
**Rapidly growing NTM**			
MABSC^*a*^	4	4	2
*M. chelonae* complex :	9	9	0
*M. chelonae*	9	9	0
*M. septicum*	1	1	0
*M. mucogenicum*	1	1	0
Total	15	15	2
**Slow-growing NTM**			
MAC (*M. avium* complex):	4	3	4
*M. avium*	4	3	4
Total	4	3	4
*Mycobacterium* spp.	1	1	0
*Mycobacteroides* spp.	1	1	0
Grand total	21	20	6
Sensitivity (%)	N/A^*b*^	95.2% (76.2%; 99.9%)	28.6% (11.3%; 52.2%)

^*a*^ MABSC included *M. abscessus* subsp. *massiliense* (*n* = 3) and *M. abscessus* subsp. *abscessus* (*n* = 1) following WGS.

^*b*^ N/A-Not Applicable.

Of the 46 NTM spiked into sputum samples from patients with cystic fibrosis, NTM Elite agar allowed the recovery of 45 NTM, i.e., a sensitivity of 97.8% [95% CI (95% CI) 88.5–99.9] compared to 16 recovered on conventional MGIT and LJ media, i.e., sensitivity of 34.8% (95% CI 21.4–50.3), including 16 NTM from MGIT [34.8% (95% CI 21.4–50.3)] and 2 NTM from LJ medium [4.4% (95% CI 0.5–14.8)] as described in Table 3.

**TABLE 3:** Nontuberculous mycobacteria from 46 contrived sputum samples recovered on NTM Elite agar and conventional mycobacterial cultures (LJ and MGIT)

	Total number of NTM recovered	28-day culture on NTM Elite agar	42- to 56-day conventional mycobacterial cultures (MGIT and LJ)
**Rapidly growing NTM**			
MABSC^*a*^	16	15	4
*M. chelonae* complex :	13	13	0
- *M. chelonae*	11	11	0
- *M. immunogenum*	2	2	0
*M. fortuitum*	1	1	1
*M. septicum*	1	1	1
Total	31	30	6
**Slow-growing NTM**			
MAC (*M. avium* complex):	11	11	9
- *M. avium*	3	3	2
- *M. intracellulare*^*b*^	8	8	7
*M. gordonae/M. paragordonae*	4	4	1
Total	15	15	10
Grand total	46	45	16
Sensitivity (%)	N/A^*c*^	97.8% (88.5%; 99.9%)	34.8% (21.4%; 50.3%)

^*a*^ MABSC included *M. abscessus* subsp. *bolletii* (*n* = 1), *M. abscessus* subsp. *massiliense* (*n* = 5), *M. abscessus* subsp. *abscessus* (*n* = 7), and three isolates unresolved to subspecies following WGS.

^*b*^ *M. intracellulare* included *M. intracellulare* subsp. *intracellulare* (*n* = 1), *M. intracellulare* subsp. *chimaera* (*n* = 4), and three isolates unresolved to subspecies following WGS.

^*c*^ N/A-Not Applicable.

NTM Elite agar allowed the growth of all species recovered during the study. Considering both fresh sputum samples and spiked NTM, the recovery rate of NTM Elite agar was significantly higher than that of conventional MGIT and LJ media combination for two rapidly growing mycobacteria groups: MABSC (19 vs 6) and *Mycobacteroides* (*Mycobacterium*) *chelonae* complex (22 vs 0). Some species were not recovered by conventional MGIT and LJ media: *M. abscessus* subsp. *bolletii*, *M. chelonae*, *Mycobacterium immunogenum*, *M. gordonae*, and *Mycobacterium mucogenicum*, as well as the un-speciated *Mycobacterium* spp. and un-speciated *Mycobacteroides* spp. isolates. Out of the 67 NTM recovered during the study from both fresh and contrived samples, two isolates were recovered by MGIT (*M. avium* and *M. abscessus* subsp. *abscessus*) that were not recovered on NTM Elite agar, and this lack of recovery was not associated with the presence of any other bacteria or fungi growing on the plates. The *M. avium* was recovered from fresh clinical sputum samples, whereas *M. abscessus* was spiked. These two isolates belonged to species recovered on NTM Elite agar for the majority of isolates, i.e., respectively, 19 out of 20 of *M. abscessus* complex and 6 out of 7 of *M. avium*.

Overall, contaminants were recovered from 17.3% (95% CI 13.4–22.2) of cultures on NTM Elite agar (49/283) against 46.3% (95% CI 40.6–52.1) on MGIT and 77.0% (95% CI 71.8–81.6) on LJ. Selectivity was defined as the proportion of the number of not contaminated samples with the method concerned (NTM Elite or the conventional MGIT and LJ media combination) out of the total number of samples. The selectivity of NTM Elite agar [82.7% (95% CI 77.8%; 86.6%)] was greater than the selectivity of the conventional MGIT and LJ media combination [18.0% (95% CI 14.0%; 22.9%)]. Contaminants from NTM Elite agar were mainly *Achromobacter* spp. (*n* = 21) and *Burkholderia* spp. (*n* = 11) as described in Table 4.

**TABLE 4:** Non-mycobacterial organisms from 283 sputum samples of 143 patients with CF recovered from NTM Elite agar^*a*^

Contaminants	Number of isolates recovered from NTM Elite agar
*Achromobacter* spp.	21
*Burkholderia* spp.	11
*Pandoraea* spp.	4
*Pseudomonas aeruginosa*	3
*Trichosporon* spp.	3
*Candida* spp.	3
*Inquilinus limosus*	2
*Delftia* spp.	2
Total	49

^*a*^ No NTM Elite agar plates were discarded from the study due to overgrowth of a non-mycobacterial organism.

### Equivalency study between NTM Elite agar and RGM medium

In the equivalency study, 27 (90%) of the NTM that were spiked into sputum samples was recovered on both NTM Elite agar and RGM medium within 28 days of incubation at 30°C ± 2°C. One strain of *M. chelonae* and one strain of *M. simiae* were not recovered on either medium. For one strain of *M. abscessus* subsp. *abscessus,* we could not confirm recovery of the spiked isolate on either medium as the un-spiked portion of the sample also produced a heavy growth of *M. abscessus* subsp. *abscessus*. The selectivity of the two media was identical as three non-mycobacterial isolates (*Pandoraea* sp., *Candida glabrata*, and *Burkholderia cepacia*) were recovered on both media from three of the 30 samples.

## DISCUSSION

NTM Elite agar, a commercially available agar, is derived from the RGM medium first described in 2016 (15) and has proven its superiority in detecting particularly rapid-growing NTM from sputum samples of CF patients. The equivalency part of the study with a collection of strains spiked into sputum samples showed identical sensitivity and selectivity between NTM Elite agar and RGM medium. The equivalency study shows supporting evidence of the equivalency of the two media which have not been published before.

This equivalent performance is confirmed in the multicenter part of this study by showing that similar performance is obtained with the industrialized version of RGM agar when compared with previously published studies with RGM medium. The sensitivity of NTM Elite agar was greater than the sensitivity of the conventional MGIT and LJ media combination for both fresh sputum samples (95.2% against 28.6%) and contrived samples (97.8% against 34.8%). This is very similar to data previously published comparing the performance of RGM or NTM Elite medium and conventional MGIT and LJ media combination with samples from pwCF (10, 11) but also with samples from people without CF (12) or samples from both (16). In the study reported by Stephenson et al. (11), a total of 130 NTM was recovered from all culture media from mostly sputum samples (99%) from the group of pwCF (*n* = 279). RGM medium sensitivity was significantly superior to the sensitivity of conventional MGIT and LJ media combination with sensitivities of 92.3% and 37.7%, respectively. In the study by Plongla et al. (10), a total of 51 NTM was recovered from 212 samples of pwCF, including 77.4% of sputum/tracheal aspirates. RGM medium showed a higher rate of recovery than conventional MGIT and LJ media combination (92.2.% vs 47.1%; *P* < 0.0001). The study by Broncano-Lavado et al. (16) included a total of 2,567 samples, mostly sputum (69%), coming from 24 hospitals from the Madrid area including four CF units. The recovery frequency, as defined by the number of recovered NTM species divided by the total number of samples, was higher with NTM Elite agar than with standard procedures (7% vs 3%; *P* < 0.001).

The observed superiority of the sensitivity of NTM Elite agar is primarily due to improved recovery of rapidly growing species (*M. abscessus* and *M. chelonae* complex) in contrast to *M. avium* complex. This observation is consistent with previous more powerful studies comparing RGM or NTM Elite media to the conventional mycobacterial cultures on pwCF (11), people without CF (12), or both (16). There is no doubt that effective recovery of *M. abscessus* is important (17). The clinical relevance, however, of *M. chelonae* isolates from respiratory tract samples is not well-defined (18).

In the equivalency study, the selectivity of NTM Elite agar and RGM medium was directly compared to each other and turned out to be identical. In the multicenter part of this study, non-mycobacterial organisms recovered on NTM Elite agar included respiratory pathogens commonly recovered in pwCF, such as *Burkholderia* spp. and *P. aeruginosa* (19). These pathogens are usually targeted by the decontamination step with the conventional MGIT and LJ media. Nonetheless, NTM Elite agar still offered a lower contamination rate than the conventional MGIT and LJ media, 17% of cultures contaminated against 46% and 77% for MGIT and LJ, respectively, in this study. The greater selectivity of NTM Elite agar would potentially lessen the workload of the laboratory. NTM Elite agar presents additional user advantages compared to the conventional MGIT and LJ media combination. This agar is easy to use as no decontamination step is required, and samples can be directly inoculated on the agar and streaked with a swab if the sample viscosity allows it. If viscosity does not allow direct inoculation, liquefaction with a dithiothreitol-based agent can be performed before inoculation. Although it is recommended to manipulate any respiratory specimen and subsequent cultures under a biosafety cabinet, mycobacteria other than those causing tuberculosis (TB), leprosy, and Buruli ulcers are not considered as Class 3 biosafety pathogens (20) and can, therefore, be cultured and manipulated in a conventional L2 microbiology laboratory. Considering the partial symptomatic overlap between tuberculosis and several other pulmonary infections, it remains recommended to screen samples and positive cultures for TB when the epidemiological or clinical contexts are suggestive of TB (21). Although the risk of exposure of laboratory staff to MTBc through NTM Elite agar manipulation cannot be fully contained in the absence of good laboratory practices described above, this risk is further reduced by the incubation temperature of this medium (30°C ± 2°C), which lies outside the optimal growth conditions of MTBc (35°C–37°C) (22) and the reduced risk of aerosol generation related to the decontamination step and the absence of manipulation of positive liquid culture media, as well as the incubation in a sealed plastic bag or a jar to avoid dehydration. It may be noted that six studies, in four countries (10–12, 16, 23, 24), have examined the culture of a cumulative total of over 5,000 respiratory samples on RGM medium/NTM Elite agar (with incubation for 28 days), and the recovery of a category 3 *Mycobacterium* species has never been reported using this medium, although the largest of these studies (16) excluded samples from patients with a clinical suspicion of tuberculosis. Laboratories are encouraged to perform their own individual risk assessment based on the types of sample they receive.

On the other hand, the multicenter study has shortcomings. First, patients’ samples were coming from three hospitals (one in Leuven, Belgium and two in Muenster, Germany), all in Europe. However, the NTM species most commonly identified in pwCF from North America and Europe are the same, i.e., *M. avium* complex (including *M. avium* and *M. intracellulare*) and MABSC. Furthermore, the results of this study (sensitivity = 95.2% vs 28.6% for conventional MGIT and LJ media combination for fresh sputum samples, and 97.8% vs 34.8% for contrived samples) are consistent with results obtained on RGM medium from pwCF in the USA (sensitivity = 92.2% vs 47.1% for conventional MGIT and LJ media combination) (10). Second, sputum samples were spiked with NTM to reach the targeted positive sample size. Nonetheless, a standard amount of NTM corresponding to the limit of detection of the NTM Elite agar was used for all spiked NTM in order to mimic real patient samples and to assess if NTM Elite agar enables the recovery of NTM in the pulmonary flora of pwCF. Also, as real sputum samples from pwCF were spiked, the potential interaction between pulmonary flora and NTM recovery was recreated. In addition, the difference from NTM Elite sensitivity to the sensitivity of the conventional MGIT and LJ media combination for contrived samples [from 97.8% (88.5%; 99.9%) to 34.8% (21.4%; 50.3%)] approximates the difference observed with fresh samples [from 95.2% (76.2%; 99.9%) to 28.6% (11.3%; 52.2%)], thus contrived samples testing reflects the fresh sputum samples testing. Third, this study compares two methods using media with different incubation temperatures (30°C ± 2°C for NTM Elite agar vs 35°C–37°C for conventional MGIT and LJ media combination); thus, the difference in incubation temperature may have also contributed to the superiority of the method using NTM Elite agar, especially for rapidly growing NTM which grow best at low temperature [28°C ± 2°C (25, 26)]. Nonetheless, even though slow-growing NTM grow best at 36°C ± 1°C (25, 26), the recovery is not very different for both methods; thus, the method using NTM Elite is sufficient enough despite non-optimal incubation temperature to allow similar recovery of slow-growing mycobacteria than with the conventional MGIT and LJ media combination. Finally, for some species, strains that were not recovered by conventional MGIT and LJ media produced very few colonies (one or two) on NTM Elite agar; thus, no strong conclusion regarding the superiority of NTM Elite agar could be drawn for these species (*M. gordonae* and *M. mucogenicum*).

### Conclusions

NTM Elite agar shows a greater sensitivity (97.0%) and selectivity (82.7%) than the conventional MGIT and LJ media combination (sensitivity = 32.8%; selectivity = 18.0%) combining MGIT and Löwenstein-Jensen. NTM Elite agar also presents user advantages compared to conventional MGIT and LJ media combination: simple and rapid sample processing without the need for an L3 biosafety laboratory as well as an earlier final reading at 28 days. NTM Elite agar being industrially produced and equivalent to RGM medium allows clinical microbiology laboratories convenient access to an easy and highly sensitive culture method for NTM. This medium is a useful adjunct to conventional AFB culture to enable the isolation of nontuberculous mycobacteria that may occasionally be killed by decontamination protocols from samples that are heavily contaminated with other bacteria and enable the culture of samples that are heavily contaminated with other bacteria (e.g., some samples from patients with cystic fibrosis).

## Data Availability

The datasets used and/or analyzed during the current study are available from the corresponding author on reasonable request.
